# IGF2BP3 May Contributes to Lung Tumorigenesis by Regulating the Alternative Splicing of PKM

**DOI:** 10.3389/fbioe.2020.00679

**Published:** 2020-09-02

**Authors:** Huang Xueqing, Zhang Jun, Jiang Yueqiang, Liao Xin, Hu Liya, Fang Yuanyuan, Zhang Yuting, Zeng Hao, Wu Hua, Liu Jian, Yin Tiejun

**Affiliations:** Department of Geriatrics, Tongji Hospital, Tongji Medical College, Huazhong University of Science and Technology, Wuhan, China

**Keywords:** lung tumorigenesis, IGF2BP3, iRIP-seq, alternative splicing, cancer therapy 3, IGF2BP3/IMP3

## Abstract

RNA binding proteins (RBPs) play a key role in genome regulation. Here we report the post-transcript regulation of IGF2BP3, which belongs to the insulin-like growth factor 2 mRNA binding protein family. We used iRIP-seq and RNA-seq to analyze the transcript regulation and alternative splicing on IGF2BP3 treated with overexpression cells and control. Overexpressed IGF2BP3 has broadly increased genes expression which involved in G-protein coupled receptor signaling pathway, positive regulation of cell proliferation, and signal transduction. IGF2BP3 regulated alternative splicing of multiple genes mainly clustered at response to hypoxia, negative regulation of transcription, and embryonic development. This study first provides alternative splicing analysis on transcription level of IGF2BP3 regulation, which laid the foundation for later research on IGF2BP3 critical functions.

## Introduction

Cancer is a complex disease caused by the malfunction of the cells regulation which is led by genetic and epigenetic mutations (Moore et al., 2018). Simply put, cancer is the abnormal growth of cells. Cancer can originate from any organ or body structure and consists of tiny cells that lose growth (Roy and Saikia, 2016). The basis of cancer hallmarks are genome unstable, which generate the genetic diversity and inflammation (Hanahan and Weinberg, 2011). Gene mutations cause cancer to be known to the public, but epigenetics is not. Epigenetic is a gene expression mutation that can be inherited but independent of DNA sequence (Kelly and Issa, 2017). Post-transcriptional gene regulation is also essential for maintaining cellular metabolism, coordinating RNA maturation, transport, stability, and degradation (Schmiedel et al., 2016).

A growing body of evidence shows that IGF2BP3 has shown its promising value on cancer therapy (Lochhead et al., 2012; Hsu et al., 2015). Numerous cancers have been identified the overexpression of IGF2BP3 including lung cancer (Findeis-Hosey and Xu, 2012), breast cancer (Scott et al., 2018), colorectal cancer (Wei et al., 2017; Xu et al., 2019), hepatocellular carcinoma (Shaalan et al., 2018), pancreatic cancer (Chen et al., 2019), glioblastoma and ovarian clear cell carcinoma (Bi et al., 2016; Dutoit et al., 2018). IGF2BP3 is a carcinoembryonic protein that is highly expressed during embryogenesis, lowly expressed in adult tissues, and re-expressed in malignant tissues (Palanichamy et al., 2016). In addition it’s the key role in fetal and adult hemoglobin expression (Tangprasittipap et al., 2018). It has been identified that IGF2BP3 can inhibit miRNA-3614 maturation thereby increased TRIM25 expression and promotes the cell proliferation of breast cancer (Wang et al., 2019). Studies showed that IGF2BP3 can induce cell proliferation and invasion through post-transcriptional regulation of IGF2 and promotes lung tumorigenesis via attenuating P53 stability which give us a hint to do more research on it (Taniuchi et al., 2014; Zhao et al., 2017). In some cases, overexpression is associated with a worse prognosis or a later stage disease (Shantha Kumara et al., 2015).

Although its function in cancer haven’t been told clearly yet, it has been investigated in other fields. IGF2BP3 (also known as IMP3) is a member of the insulin-like growth factor 2 mRNA binding protein family (Schmiedel et al., 2016). IGF2BP3 is a 580 amino acid protein which has two RNA recognition motifs and four K homology domains, which was encoded by a 4350-bp mRNA transcript generated by the IGF2BP3 gene on chromosome 7p11.5 (Shantha Kumara et al., 2015). RNA binding protein (often abbreviated as RBP), as a crucial molecule, involves in almost all stages of post-transcriptional regulation, thus determining the destiny and function of each transcript in the cell and ensuring cell homeostasis (Lukong et al., 2008; Pereira et al., 2017). RBP, can be classified by the ability of binding to all transcripts (usually by common RNA elements) or recognizing specific transcripts (by specific motifs) (Rissland, 2017). Over the past few years, RBPs have been discovered serve as a hub molecule and identified to be hallmarks in more than one cancer (Lukong et al., 2008; Gerstberger et al., 2014; Brinegar and Cooper, 2016). RBPs also. RBPs involved in all aspects of RNA metabolism and play a pivotal role in cancer through a wide range of mechanisms, including genomic alterations, and post-transcriptional control (Fu and Ares, 2014; Mitchell and Parker, 2014; Brinegar and Cooper, 2016). Therefore, understanding the mechanisms by which RBP regulate cancer development is critical to reducing its morbidity and mortality. Since alternative splicing (AS) is a mechanism of post-transcriptional RNA processing that affords a significant evolutionary superiority by producing multiple mRNAs during pre-mRNA maturation, resulting in the production of multiple proteins with different functions from a single gene (Le et al., 2015; Brander et al., 2018; Liu et al., 2018). Accordingly, we hypothesized IGF2BP3 may also play a role in alternative splicing.

Herein, we aimed at the potential role of IGF2BP3 in mRNA binding and alternative splicing in A549 cells and its regulatory functions. We used RIP-seq technology to capture IGF2BP3-bound RNAs and their interaction sites. The results indicated IGF2BP3 has a good capability on RNA binding and strongly associates with mRNAs more at CDS regions than at 3′UTRs. In line with these, we performed high-throughput RNA sequencing (RNA-seq) for overexpressed IGF2BP3 and control cells to investigate the impact of IGF2BP3 on gene expression levels. The results showed that there are distinct changes in transcript profiles and IGF2BP3 strongly associates with mRNAs more at 5′UTR regions than at 3′UTRs. Then we delve into the role of IGF2BP3 in alternative splicing. Totally, we identified 11,418 alternative splicing events (*p*-value cutoff <0.05), including 6,804 novel alternative splicing events (RASEs) and 61 IGF2BP3-bound and –regulated alternative splicing genes were identified. We also found the alternative splicing pattern of the PKM gene, a kinase of pyruvate and its splicing pattern highly expressed in lung cancer cells (Mi et al., 2017), which is regulated by IGF2BP3. Taken together, our work clearly identified a complex genome-wide IGF2BP3-RNA interaction map in human lung cancer cell line and indicated that IGF2BP3 binds to CDS regions and regulates multiple alternative splicing events, which will help to understand the regulatory mechanisms of IGF2BP3 at the pre-RNA splicing level and provide insight into the role of IGF2BP3 in multiple biological processes.

## Results

### IGF2BP3 Expression Level Was Significantly Increased in Multiple Cancer Types

**FIGURE 1:**
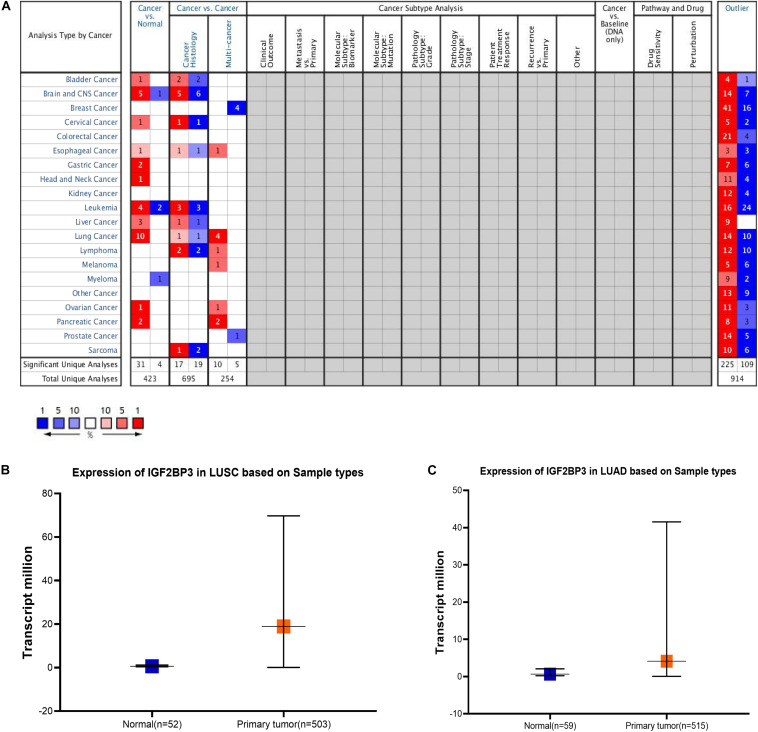
IGF2BP3 expression level was significantly increased in multiple cancer types. **(A)** Different expression of IGF2BP3 in multiple cancer types. The data was downloaded from Oncomine website. **(B,C)** Plot of the expression of IGF2BP3 in normal and LUAD and LUSC samples. The data was downloaded from TCGA website.

According to previous studies, IGF2BP3 is highly expressed in numerous cancer tissues, such as esophageal cancer, lung cancer, prostate cancer, gastric cancer, and colorectal malignancies (Shantha Kumara et al., 2015; Zhang et al., 2017). At the tissue level, previous study has shown IGF2BP3 is higher expressed in squamous cell carcinoma and adenocarcinoma (Zhao et al., 2017). So we first studied the expression level of IGF2BP3 in different cancer types through the Oncomine database (Figure 1A). In the grid in the center of the table, blue indicates low expression of IGF2BP3 in the corresponding tumor, red indicates high expression, and gray indicates no data. And the numbers in the table represent the number of studies that meet the screening criteria which *P*-value is 1E-5 and the fold change is 3. Then, we found that IGF2BP3 is highly expressed in lung cancer tissues and low in normal lung tissues (Figures 1B,C). Taken together, these results indicate that IGF2BP3 is highly expressed in multiple cancer tissues, especially in lung squamous cell carcinoma and lung adenocarcinoma.

### IGF2BP3 Strongly Associates With mRNAs More at 5′UTR Regions and 3′UTR Regions

**FIGURE 2:**
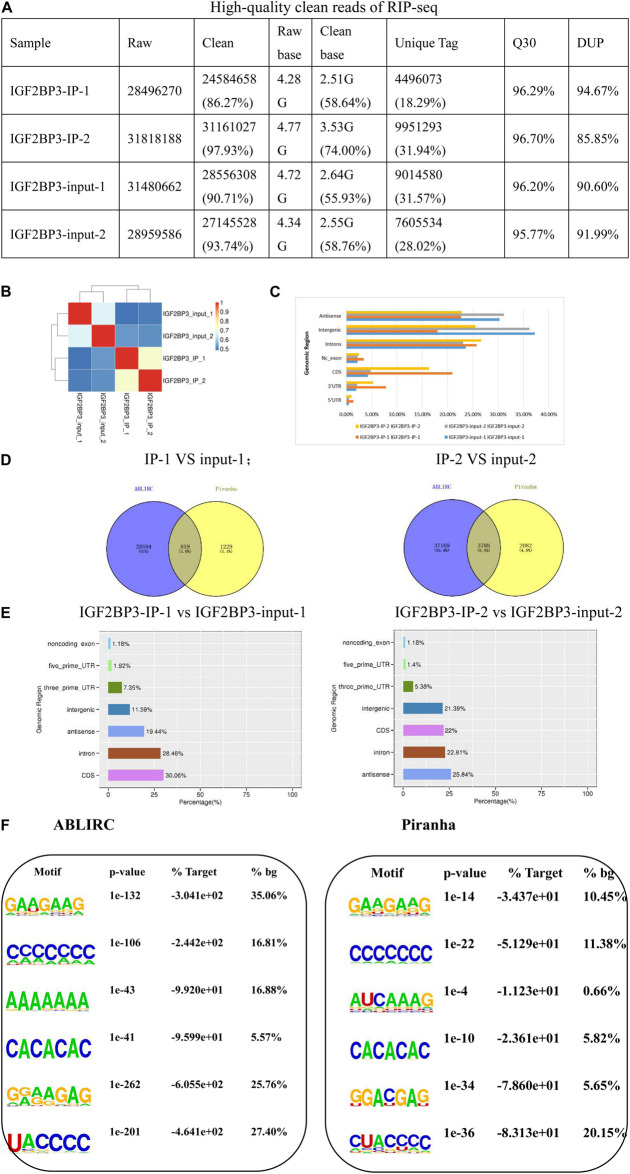
IGF2BP3 strongly associates with mRNAs more at CDS regions than at 3′UTRs. **(A)** The form shows the quality control of our iRIP-sequence is good. (1) Raw data: the number of original sequences transformed from the original image data obtained by sequencing through base calling; (2) Clean reads: the raw reads are stripped of the adapter sequence, and the number of valid sequences obtained after low-quality bases is used for subsequent analysis; (3) Raw base: the count the number of bases it contains, based on the number and length of raw reads, in G; (4) Clean base: according to the number and length of clean reads, count the number of bases it contains, in G; (5) Unique tag: unique tag, the number of non-repeating reads and its proportion of clean reads; (6) Q30: Proportion of bases whose sequencing error rate is less than 0.1%; (7) DUP: duplication level. The ratio of duplicate reads to total reads. **(B)** Heat map showing the hierarchically clustered Pearson correlation matrix resulting from comparing the transcript expression values for control and IGF2BP3 IP samples. **(C)** Bar plot of the genomic region distribution of the control and uniquely mapped IGF2BP3 IP reads. **(D)** The peak distribution across reference genomic region of the IGF2BP3 IP and control group. **(E)** Venn diagram analysis from the comparative result of ABLIRC and Piranha peak calling methods. **(F)** Extracted IGF2BP3 peaks motifs using ABLIRC or Piranha.

To our knowledge, a classic RBP is evaluated by its ability of binding and the amount of bound-reads, as well as its efficiency. For these reasons, we attempt to explore IGF2BP3’s binding ability by using RNA Immunoprecipitation (RIP) method. We used the improved RIP and high throughput sequencing approach (iRIP-seq, see “Materials and Methods for detail information) to identify the transcripts which are interacting with IGF2BP3 in A549 cells. As shown in Figure 2A, we can see that the quality control of iRIP-sequence is good. Then, we utilized Hi-seq 2000 platform to sequence the cDNA libraries of RNAs from anti-IGF2BP3 and IgG immunoprecipitates. After removing the reads of low-quality and adaptor sequences, IGF2BP3 and IgG immunoprecipitates totally recovered 27,872,842 and 27,850,918 reads. It turned out that there were about 39.58% reads can align when we use these data to locate to the GRCh38 genome by using the Tophat2 (Supplementary Table S1). Due to the existence of broken adaptor and primer sequences, there were a lot of unaligned reads. Most anti-IGF2BP3 reads can be uniquely mapped, however, many fewer IgG reads were either uniquely mapped or mapped.

To gain insight into the particularity of IGF2BP3 pull-downs, IgG was set as the blank control of immunoprecipitation. To assure the reliability of the results, we conducted two reduplicative experiments. As shown in Figure 2B, the correlation of IGF2BP3 samples are high and obvious different with the control. It is worth noting that the results of the two tests are almost identical, indicating that the sample is reproducible. Collectively, these results demonstrate the ability of IGF2BP3 samples to bind to RNA has a good specificity. Usually, in CLIP-seq protocols, ribonuclease was used to develop short-length reads from the sites that protected by RBPs, whose function is to digest the immunoprecipitated RNAs from the RBP-RNA complexes (Xue et al., 2009). But in RIP protocols, there was no RNase digestion step and doesn’t recover intact transcripts. Surprisingly, the results showed that when the distribution of uniquely mapped anti-IGF2BP3 reads were potted on the whole human genome, the RIP-seq reads were much higher enriched in 5′UTR regions than in 3′UTR regions, and also enriched in CDS regions (Figure 2C).

From what we know, a gene with a low transcript abundance is less likely to be caught by an RNA-binding protein during IP (Chi et al., 2009). Recent days, we noticed that there is a software tool, ABLIRC, which can be used to recover the IGF2BP3 binding sites from the RIP-seq reads. Based on the results identified by the ABLIRC algorithm, we observed 77.36% sense peaks and 22.64% anti-sense peaks from IGF2BP3-associated RNAs and control (Figure 2D). After filtered the overlapping peaks of IGF2BP3 and IgG samples, 11,238 genes were distributed by a total of 38,608 (sense and antisense) peaks. The number of intronic peak in IGF2BP3 sample is in significantly contrast to the IgG sample. To verify these conclusions, we used Piranha, a published software to identify the RNA-protein interaction sites from the data of high-throughput sequencing, to call IGF2BP3-bound peaks. As shown in Figure 2E, Piranha average validated 2,303 peaks from ABLIRC averagely, representing 7.39% and 58.18% of ABLIRC and Piranha peaks, correspondingly. Furthermore, we found that 5′ss motif, 3′ss motif, and GA-rich motif were high enriched in Piranha peaks (Figure 2F), which was likely with those in ABLIRC peaks.

In summary, we successfully extracted the binding motif of IGF2BP3 RNA-binding protein by ABLIRC algorithm, which will help us understand the comprehensive regulation of IGF2BP3-RNA interaction during gene expression of A549 cells.

### IGF2BP3-Bound Genes Are Distributed in Various Pathways

**FIGURE 3:**
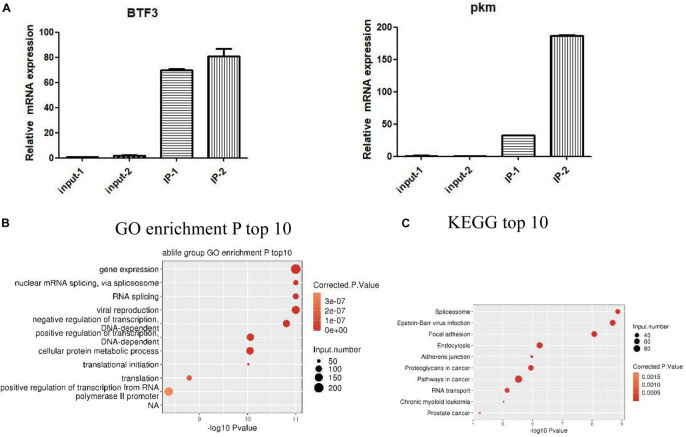
IGF2BP3-bound genes are distributed in various pathways. **(A)** RIP-qPCR validation of IGF2BP3-bound genes. **(B,C)** The top 10 representative pathways of IGF2BP3-bound genes in GO database and KEGG database.

We identified IGF2BP3 binding genes in A549 cells by ABLIRC. Supplementary Table S2 lists a total of 11,238 genes were detected with IGF2BP3 binding signal by our iRIP-seq data. As shown in Supplementary Table S3, we can see the ranks of candidates of IGF2BP3 targets, such as KRT7, PTMS, PKM, and IFI6. In order to investigate more functions of IGF2BP3, we selected some IGF2BP3 binding targets for RIP-PCR validation. According to the results of the quantitative RIP-PCR, we found that all candidate RNA targets were significantly enriched with anti-IGF2BP3 immunoprecipitates relative to the IgG control group (Figure 3A). For example, BTF3 relatives to IgG immunoprecipitates, which have been identified as previous IGF2BP3s’ targets, were observed significantly enriched in IGF2BP3 immunoprecipitates. And we also found that HNRNPDL RNA was just enriched in one group of IGF2BP3 immunoprecipitate. Furthermore, we noticed that PKM RNA, which can generate a marker gene PKM2 for lung cancer (Rzechonek et al., 2017), is efficiently enriched in IGF2BP3 immunoprecipitates. We then assigned the identified peak genes of IGF2BP3 targets to the pathways in the Gene Ontology database and KEGG database, resulting in 867 GO biological processes and 289 KEGG biochemical pathways (Supplementary Dataset 1). The top 10 pathways are shown in Figures 3B,C. As shown in Figure 3B, IGF2BP3 involved in gene expression, nuclear mRNA splicing which via spliceosome and RNA splicing activities. On the other hand, based on KEGG annotation, we find that the spliceosome pathway ranks first and IGF2BP3 targets also involved in the processes like focal adhesion, proteoglycans in cancer, pathways in cancer, and RNA transport. Therefore we hypothesis IGF2BP3 has a strong regulation on the alternative splicing process.

### IGF2BP3 Regulates Multiple Alternative Splicing Events

**FIGURE 4:**
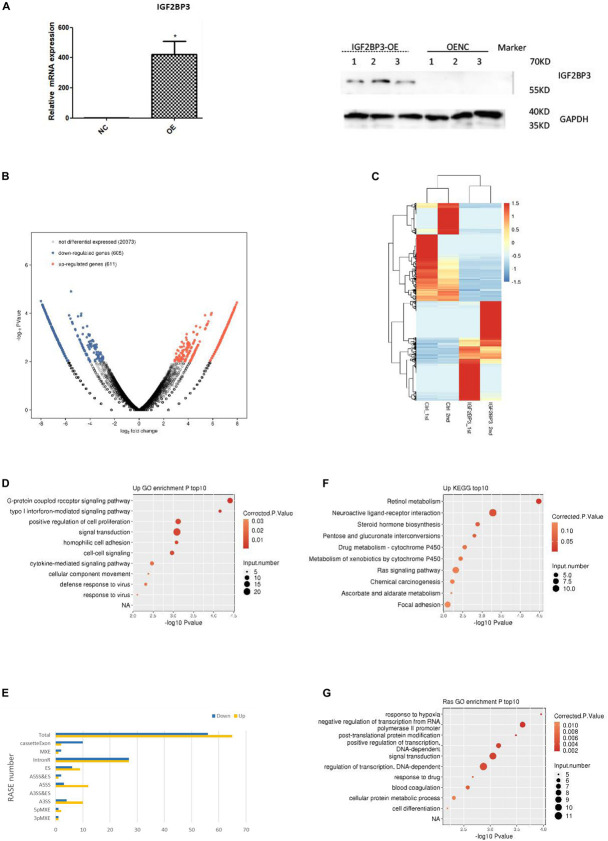
IGF2BP3 regulates multiple alternative splicing events. **(A)** IGF2BP3 expression on mRNA and protein level in A549 cells after transiently transfected with IGF2BP3 specific overexpression RNA or control vector, as determined by qRT-PCR and Western blot analysis. **(B)** Total number of differentially expressed genes. **(C)** Hierarchical clustering of DEGs in overexpressed and control samples. Expression values (FPKM) are log2-transformed and then median-centered by each gene. **(D,E)** The top 10 representative GO Biological Process terms of IGF2BP3-regulated genes. **(F)** Classification of different AS types regulated by IGF2BP3 protein. **(G)** The top 10 GO Pathways for gene aggregation of differential AS events.

As the research progressed, we decided to explore the potential function of IGF2BP3 in A549 cells by using transcriptome sequencing (RNA-seq) with two biological replicates. We first examine the quality of data which shows in the Supplementary Table S4. As shown in Figure 4A, quantitative PCR and Western blot experiments both confirmed that the expression of IGF2BP3 was significantly increased in transfected A549 cells. The genes expressed differentially was identified by using edgeR (Robinson et al., 2010). Interestingly, we identified that among all expressed genes, there are 611 up-regulated and 605 down-regulated genes, which indicate that IGF2BP3 regulates gene transcription broadly (Figure 4B). We can found the details of the differentially expressed genes (DEGs) in Supplementary Table S5. As shown in the heatmap analysis based on the expression patterns of DEGs which in RNA-seq samples, there are a high consistency of the IGF2BP3-regulated transcription in both sets (Figure 4C). To further characterize the potential biological functions of these DEGs, DEGs resulted from whole expressed genes were subjected to GO and KEGG database. As shown in Figure 4D, shows several pathways which up-regulated genes annotated with categories of GO, including G-protein coupled receptor signaling pathway, positive regulation of cell proliferation and signal transduction. In line with these, the up-regulated genes annotated with KEGG categories in A549 cells are associated with retinol metabolism, neuroactive ligand-receptor interaction and Ras signaling pathway (Figure 4E).

Since one key aim of our study was to investigate the role of IGF2BP3 on alternative splicing (AS) regulation, thus we used transcriptome sequencing to analyze the AS events of IGF2BP3-dependent in A549 cells. The first transcriptome was generated by sequencing 47,749,031 in the original data, there is 44.38% is the junction reads. After analysis by the ABLAS tool, 9745 alternative splicing events (ASE) were obtained. These results enlarged the role of IGF2BP3 on the regulation of alternative splicing. In line with these, we performed a deeper transcriptome sequencing to see the distribution sites of alternative splicing events. Figure 4F showed that 121 AS events which changed significantly have been detected. Among them, a lot of splicing events belonged to Intron R (intron retention, 54), A5SS (alternative 5′ splice site, 15), ES (exon skipping, 15), and A3SS (alternative 3′ splice site, 14) categories. The other IGF2BP3-bound and IGF2BP3–affected splicing events included 3pMXE (mutually exclusive exons, 2), 5pMXE (mutually exclusive 5′ UTRs, 3), and Cassette Exon (number: 12). Taken together, we can know that Intron R, A5SS, ES are main splicing events regulated by IGF2BP3, which indicated that IGF2BP3 regulates alternative splicing events all over in A549 cells.

To further investigate the relationship between IGF2BP3 binding and the regulation of alternative splicing, we performed overlap analysis of the DEGs from the overexpressed RNA-seq analysis and the peak genes from the iRIP-seq analysis. The results showed that there were 61 overlap genes. Next, we performed functional clustering of genes with both binding and alternative splicing. The results showed that the above genes are mainly clustered at response to hypoxia, negative regulation of transcription, and embryonic development (Figure 4G).

### IGF2BP3 Administered the Alternative Splicing of PKM

**FIGURE 5:**
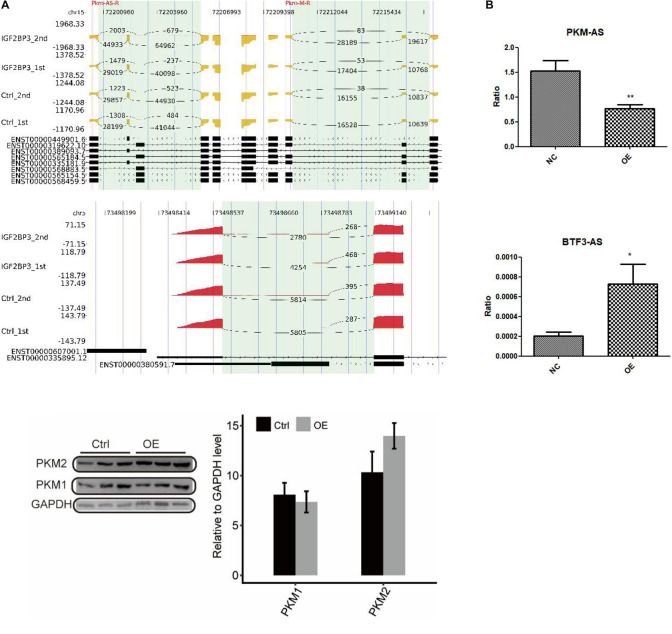
Validation of IGF2BP3-affected AS events. **(A)** The distribution of reads across the whole region of PKM and BTF3. **(B)** RT-qPCR validation of PKM and BTF3 ASEs regulated by IGF2BP3.

To directly assess the role of IGF2BP3 in these alternative splicing events, we undertook an RT-PCR experiment to examine the changes of the type of alternative splicing between control and A549 cells with overexpressed IGF2BP3. The inclusion/exclusion (In/Ex) ratio was used to measure the quantification of AS pattern. Figure 5A and Supplementary Datasets 2,3 shows some representative affected AS events which has two alternative splicing patterns, including intron retention and alternative 5′splice site. Among those AS genes, PKM and BTF3 came into our notice, whose dysregulation associated with cell proliferation (Dayton et al., 2016). In this study, we first used RIP-seq protocol to analyze the interaction between IGF2BP3 and PKM pre-mRNA. As shown in the Figure 5 (left panel), there are a large amount of IGF2BP3-bound peaks located in the exonic position. These two experiments’ results both implied that PKM is a direct target of IGF2BP3 protein in A549 cells.

To gain insight into the regulation of alternative splicing events of PKM and BTF3 whether regulated by IGF2BP3-bound, we used Q-PCR to investigate the changes of ASEs in A549 cells with overexpressed IGF2BP3. As shown in Figure 5B, the ASEs of PKM is showing a decrease trend, whereas BTF3 showed an increase. Taken together, our results indicated that in A549 cells alternative splicing of PKM and BTF3 are regulated by IGF2BP3 directly.

## Discussion

Being a significant RBP, IGF2BP3 is involved in the process of mRNA metabolism and contributes to the cell proliferation and invasion in various cancers (Samanta et al., 2012; Taniuchi et al., 2014; Hsu et al., 2015; Palanichamy et al., 2016; Zhang et al., 2017; Xu et al., 2019). Moreover, multiple evidences proved that IGF2BP3 is relate to the processes of cancer development and prognosis (Lochhead et al., 2012; Cao et al., 2018). Given these foundations, some researches have been performed to comprehensively understand the functions of IGF2BP3 (Cao et al., 2018; Mancarella et al., 2018; Wang et al., 2019).

Herein, we used iRIP-seq to uncover the mysteries of interactions between IGF2BP3 and RNA in human cancer cell line. The results of RIP-seq showed that sequencing reads of IGF2BP3-associated RNAs were mostly mapped to intron region, CDS region and intergenic region, which indicated IGF2BP3 has functional RNA targets *in vivo*. Of note, about 21.175% reads were allocated on intergenic region, which generate microRNA and other kinds of non-coding RNA. This result provide us a new hint that IGF2BP3 might participate in the process of non-coding RNA metabolism, which has never been reported and would be worthy to further study.

In here, as for the analysis of our iRIP-seq results, we utilized the ABLIRC to analyze the abundant peaks of IGF2BP3, which showed a significant fraction of IGF2BP3 binding peaks were enriched at 5′UTR region, 3′UTR region, and CDS. To be specific, IGF2BP3 might regulate the splice junction via the recognition of 3′ UTR region and 5′UTR region in A549 cells. The recognition of 3′UTR region and 5′UTR region in pre-mRNA are essential to the alternative splicing events. By using the iRIP-PCR, we verified that BTF3, PKM, and HNRNPDL are IGF2BP3-bound targets. From the GO and KEGG results we’ve got, IGF2BP3 involved in various important pathways, including gene expression, RNA splicing activities, and spliceosome pathway which ranks first. Therefore we speculated that IGF2BP3 can regulate alternative splicing in A549 cells. These observations might broad the understanding of the mechanism in the pre-mRNA alternative splicing regulation of IGF2BP3. We then used RNA-seq to analyze the alternative splicing events regulated by IGF2BP3. We have identified 4614 RASE from 11418 alternative splicing events. We noticed that almost half of the pre-mRNA targets are regulated via directly binding to the IGF2BP3 in A549 cells. Therefore, we concluded that IGF2BP3 involved in different kinds of alternative splicing events regulation, such as NFKB2, LUC7L, BTF3, and PKM.

In the end, we speculated that PKM pre-mRNA may act as a new target of IGF2BP3. Our results indicated that the overexpression of IGF2BP3 has a regulation on the alternative splicing of PKM and BTF3. Very importantly, PKM is universally expressed in cancer and play a pivotal role in maintaining the metabolic program during cancer progression (Yang and Lu, 2013). In addition, PKM2 has been viewed as a potential target of lung cancer and its activity has been found higher in NSCLC patients than in normal subjects (Mi et al., 2017; Rzechonek et al., 2017).

We know that the final rate-limiting step in glycolysis is regulated by pyruvate kinase (PK) and catalyzes the transfer of phosphate groups from phosphoenolpyruvate (PEP) to adenosine diphosphate (Yang and Lu, 2013). As we know, PKM2 is an M2 isoform of PK, together with PKM1, is encoded by PKM. PKM pre-mRNA is spliced alternatively by the poly pyrimidine-tract binding (PTB) protein splicing factors and the heterogeneous nuclear ribonucleoproteins (hnRNPs) A1/2. As a result, PKM1 and PKM2 are generated by the exclusion of exon 9 and the inclusion of exon 10. Through the process of retaining exon 10, PKM2 owns unique properties which is important in the reprogramming of cell metabolism (Wong et al., 2015). PKM2 can be detected in normal tissues such as lung, liver, and kidney and also can expressed in cells with a high rate of nucleic synthesis. The expression of PKM2 is regulated at multiple levels by the regulation of DNA methylation, pre-mRNA splicing of PKM and post-translational modifications of the PKM2 protein (Yang and Lu, 2015).

In most cancer cells, the expression of PKM2 is increased and the expression of PKM2 in cancer cells affects aerobic glycolysis (Dayton et al., 2016). This phenomenon known as Warburg effect which tell the special way of energy production of tumor cells is very special: most tumor cells pass relatively low yields of glycolic acid to itself, whereas this kind of mechanism of action neither require oxygen nor mitochondria involvement (Koppenol et al., 2011). Recent studies showed that the replacement from PKM2 to PKM1 has been verified the ability to suppress aerobic glycolysis and tumor growth. PKM2 also can regulate tumor formation and growth by acting in gene transcription (Guo et al., 2017). It has also been proved that the splicing of PKM2 exist in drug resistance-pancreatic ductal adenocarcinoma (Calabretta et al., 2016). These evidence positions PKM2 as a promising target for cancer therapy. Aggregating our results, our observation might offer a new evidence about the mechanisms of how IGF2BP3 having an impact on lung cancer.

For the first time, we have demonstrated IGF2BP3 regulation of alternative splicing by successfully applied RIP-seq and RNA-seq in A549 cell line. Although we have identified the direct regulation in alternative splicing between IGF2BP3 and PKM, the exact mechanism by which IGF2BP3 regulate the alternative splicing of PKM has not been completely elucidated, which needs further investigations. Collectively, our work paves the way for seek the new biological function in the progress of tumorigenesis and provide new clues of the target therapy in lung cancer.

## Materials and Methods

### Cell Culture and Transfections

A549 cells were cultured under standard conditions with Dulbecco’s modified Eagle’s medium (DMEM) with 10% fetal bovine serum (FBS), 100 μg/mL streptomycin, and 100 U/mL penicillin.

According to the manufacturer’s instructions, the transfection of A549 cells with an IGF2BP3-overexpressing plasmid was performed using Lipofectamine 2000 (Invitrogen, Carlsbad, CA, United States). After 48 h, q-PCR and western blot assay were performed to confirm the overexpression of IGF2BP3 mRNA and protein expression level (Tu et al., 2019).

### Western Blotting Analysis

We loaded protein samples into 10% or 12% SDS-PAGE gels depending on molecular weight and transferred them onto 0.45 mm PVDF membranes. Then the PVDF membranes were blocked with 5% skim milk (in a buffer containing 10 mM Tris, pH 8.0, 150 mM NaCl, 0.05% Tween 20) for an hour. Next we incubated overnight with primary antibody at 4°C and later incubated with horseradish peroxidase-conjugated secondary antibody for 1 h at room temperature. Then, through the chemiluminescence, membranes were visualized. We also have quantitated some of the WB bands by the software Image J. Antibodies: The following antibodies were purchased from commercial sources from ABlife Company.

### iRIP-seq Library Preparation and Sequencing

The IGF2BP3-binding RNAs were isolated by using TRIzol (Invitrogen). Complementary DNA (cDNA) libraries were drew up with the ScriptSeq RNA-seq Library Preparation Kit (SSV21124, Illumina). The libraries were prepared on the basis of the manufacturer’s instructions and used to the Illumina HiSeq X Ten system for 150 nt paired-end sequencing.

### iRIP-seq Peak Calling Analysis

After we aligned reads onto the genome, only uniquely mapped reads were applied for the following analysis. “ABLIRC” strategy was applied to identify the binding regions of TTP on genome (Xia et al., 2017). At least 1 bp overlapped reads were clustered as peaks. For each gene, we used computational simulation to randomly produced reads with the same number and lengths as reads in peaks. The outputting reads were further mapped to the same genes to generate random max peak height from overlapping reads. The whole process was repeated for 500 times. All the observed peaks with heights higher than those of random max peaks (*p*-value < 0.05) were selected. The IGF2BP3 and Input samples were analyzed by the simulation independently, and the IGF2BP3 peaks that have overlap with Input peaks were removed. The target genes of IGF2BP3 were finally determined by the peaks and the binding motifs of IGF2BP3 protein were called by Homer software (Heinz et al., 2010).

### RIP-qPCR

We used TRIzol (Invitrogen) to extract total RNAs from the immunoprecipitate of IGF2BP3 based on the manufacturer’s instructions. Random primer was used for the cDNA synthesis. To detect whether IGF2BP3 target genes were significantly and specifically enriched in the IGF2BP3 immunoprecipitate, we then used normal PCR as a validation. At the meantime, we used input RNA as a reference and performed quantitative RT-PCR as characterized to determine the relative level of specific RNAs in the IGF2BP3 immunoprecipitates and IgG (Zhao et al., 2010). Q-PCR data represents the mean values from at least three independent experiments. Several genes were selected for PCR-amplification both in IGF2BP3 and IgG immunoprecipitates.

### RNA Extraction and High-Throughput Sequencing

Total RNA was extracted by the TRIZOL (Ambion) and was purified with two phenol-chloroform treatments later. In order to remove DNA, the purified RNA was treated with RQ1 DNase (RNase free) (Promega, Madison, WI, United States) and its quality and quantity were reassured by measuring the absorbance at 260 nm/280 nm (A260/A280) using Smartspec Plus (Bio-Rad, United States). The integrity of RNA was then confirmed by 1.5% agarose gel electrophoresis. 10 μg of the total RNA for each sample was used to preparing directional RNA-seq library. Ahead of that, the polyadenylated mRNAs were condensed with oligo (dT)-conjugated magnetic beads (Invitrogen, Carlsbad, CA, United States). Next, the concentrated mRNAs were iron fragmented at 95°C, end repaired and 5′ adaptor ligated with 5′ adaptor. In line with it, we performed reverse transcription (RT) with RT primer harboring 3′ adaptor sequence and randomized hexamer. Before they were used for sequencing, the purified cDNAs were amplified and stored at −80°C (Li et al., 2019). As reported by the manufacturer’s instructions, the libraries were ready for high-throughput sequencing. We used Illumina HiSeq4000 system to collect data from 151-bp pair-end sequencing (ABlife Inc., Wuhan, China).

### RNA-seq Raw Data Clean and Alignment

We first discarded raw reads containing more than 2-N bases. Then we trimmed adaptors and low quality bases from raw sequencing reads using FASTX-Toolkit (Version 0.0.13). The short reads less than 16 nt were dropped too. Following that, clean reads were aligned to the GRch38 genome by TopHat2 (Kim et al., 2013) with 4 mismatches. Uniquely mapped reads were applied to calculate reads number and FPKM value (FPKM represents fragments per kilobase and per million) for each gene.

### Differentially Expressed Genes (DEG) Analysis

FPKM (paired-end fragments per kilobase of exon per million fragments mapped), was used to assess the expression level of genes. In order to screen out the DEGs, we used the software edgeR (Robinson et al., 2010), which was specifically used for analyzing the differential expression of genes by using raw RNA-seq reads.

So as to determine whether a gene was differentially expressed, we analyzed the results on the basis of the fold change (fold change ≥2 or ≤0.5) and false discovery rate (FDR < 0.05). To gain insight into the gene function and measure the functional category distribution frequency, Gene Ontology (GO) analyses and enriched KEGG pathway were analyzed using KOBAS 2.0 server (Xie et al., 2011). We used Hypergeometric test and Benjamini-Hochberg FDR controlling procedure to define the enrichment of each pathway (corrected *p*-value < 0.05).

### Alternative Splicing Analysis

As described previously, the ABLas pipeline (Jin et al., 2017; Xia et al., 2017) was used to define and quantify the ASEs (alternative splicing events) and RASEs (regulated alternative splicing events) between the samples. In short, detection of seven types of canonical ASEs in each sample was on the basis of the splice junction reads. These ASEs were exon skipping (ES), cassette exon (cassette Exon, CE), alternative 3′splice site (A3SS), alternative 5′splice site (A5SS), mutual exclusive exon skipping (MXE), the MXE combined with alternative polyadenylation site (3pMXE), and with alternative 5′ promoter (5pMXE). Then, the significant *p*-value was calculated by fisher’s exact test, with the model reads of samples and alternative reads as input data, separately. RASE ratio was defined by the changed ratio of alternatively spliced reads and constitutively spliced reads between compared samples. The RASE ratio >0.2 and *p*-value < 0.05 were set as the threshold for RASEs detection.

### Reverse Transcription qPCR Affirmation of Alternative Splicing Events

To exemplify the validity of ASEs in A549 cells, quantitative reverse-transcription polymerase chain reaction (RT-qPCR) was used in some selected RASEs, and standardized with the reference gene GAPDH. The primers which were used for detecting the pre-mRNA splicing are shown in Supplementary Table S6. In order to quantitatively analyzing the two different splicing isoforms of a specific ASE using a qPCR approach, we arranged two pairs of primers to specifically amplify each of these two isoforms after the initial synthesis of the first strand cDNA using random primers. To achieve this specificity, we designed a primer which can pair the splice junction of the constitutive exon and alternative exon. The RNA samples used for RT-qPCR and for RNA-seq too. The PCR conditions included denaturing at 95°C for 10 min, and 40 cycles of denaturing at 95°C for 15 s, then annealing and extension at 60°C for 1 min. PCR amplifications were performed in triplicate for control and IGF2BP3-OE samples, respectively.

### Statistical Analysis

All the statistics were showed as mean ± standard deviation (SD) and processed by SPSS 16.0 statistical software (Chicago, IL, United States). All experiments were operated at least three times independently, and values of *P* < 0.05 were considered considerable.

## Data Availability Statement

The raw data supporting the conclusions of this article will be made available by the authors, without undue reservation, to any qualified researcher.

## Author Contributions

YT and ZJ conceived the project. HX, FY, ZY, and ZH implemented the experiments and analyzed the data. ZH, HX, ZJ, and JY prepared the data and performed literature search. WH, LJ, and HL guided the experimental plan and writing of the subject. HX wrote the manuscript. All authors approved the final manuscript.

## Conflict of Interest

The authors declare that the research was conducted in the absence of any commercial or financial relationships that could be construed as a potential conflict of interest.
