# Supplementary figures and images for: IGF2BP3 May Contributes to Lung Tumorigenesis by Regulating the Alternative Splicing of PKM
Source: Front Bioeng Biotechnol. 2020 Sep 2;8:679. doi: 10.3389/fbioe.2020.00679 (PMC7492387; doi:10.3389/fbioe.2020.00679)

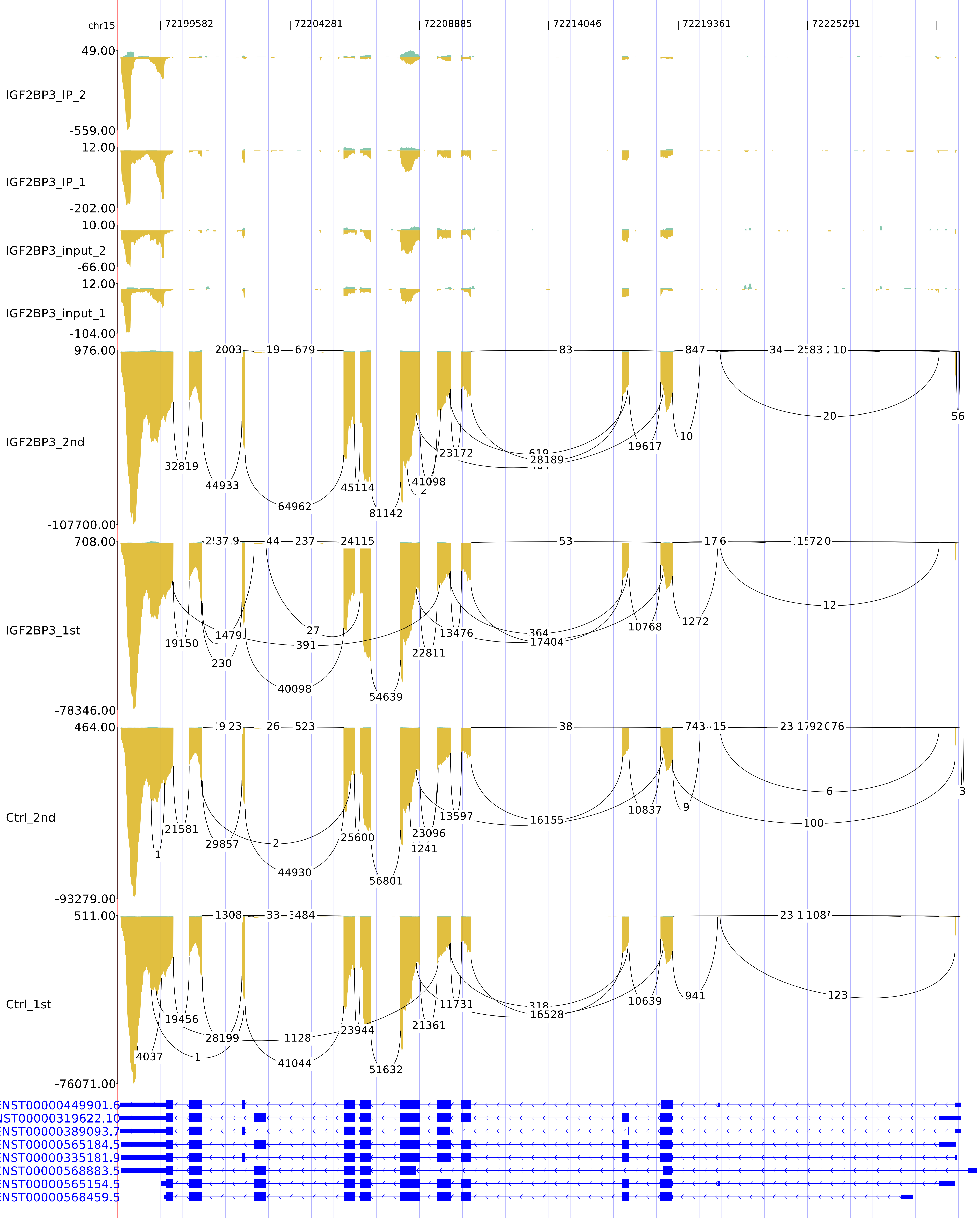

Supplement: Supplementary file 8 [file Data_Sheet_2.zip › ENSG00000067225.17_PKM.pdf]

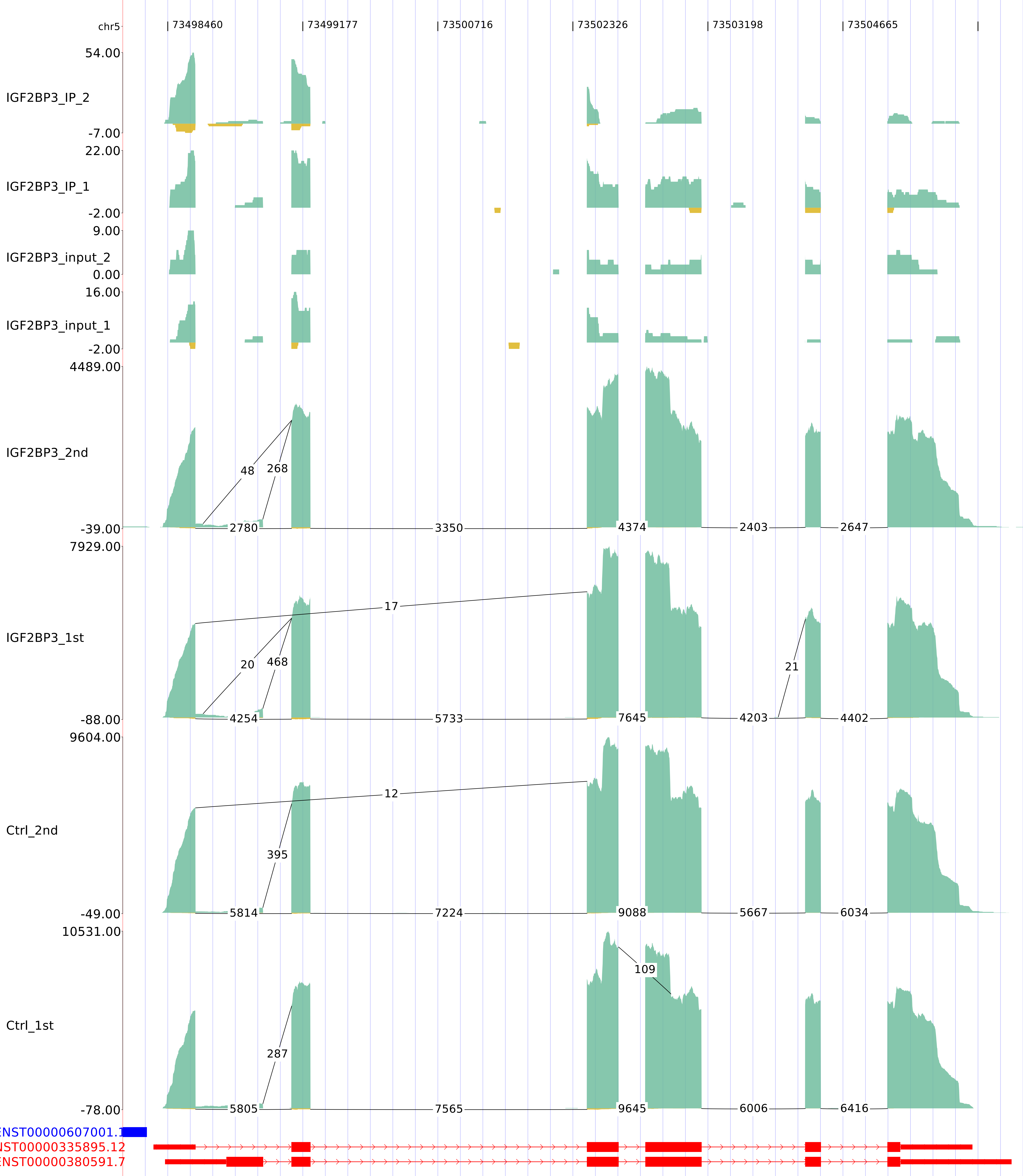

Supplement: Supplementary file 8 [file Data_Sheet_2.zip › ENSG00000145741.15_BTF3.pdf]

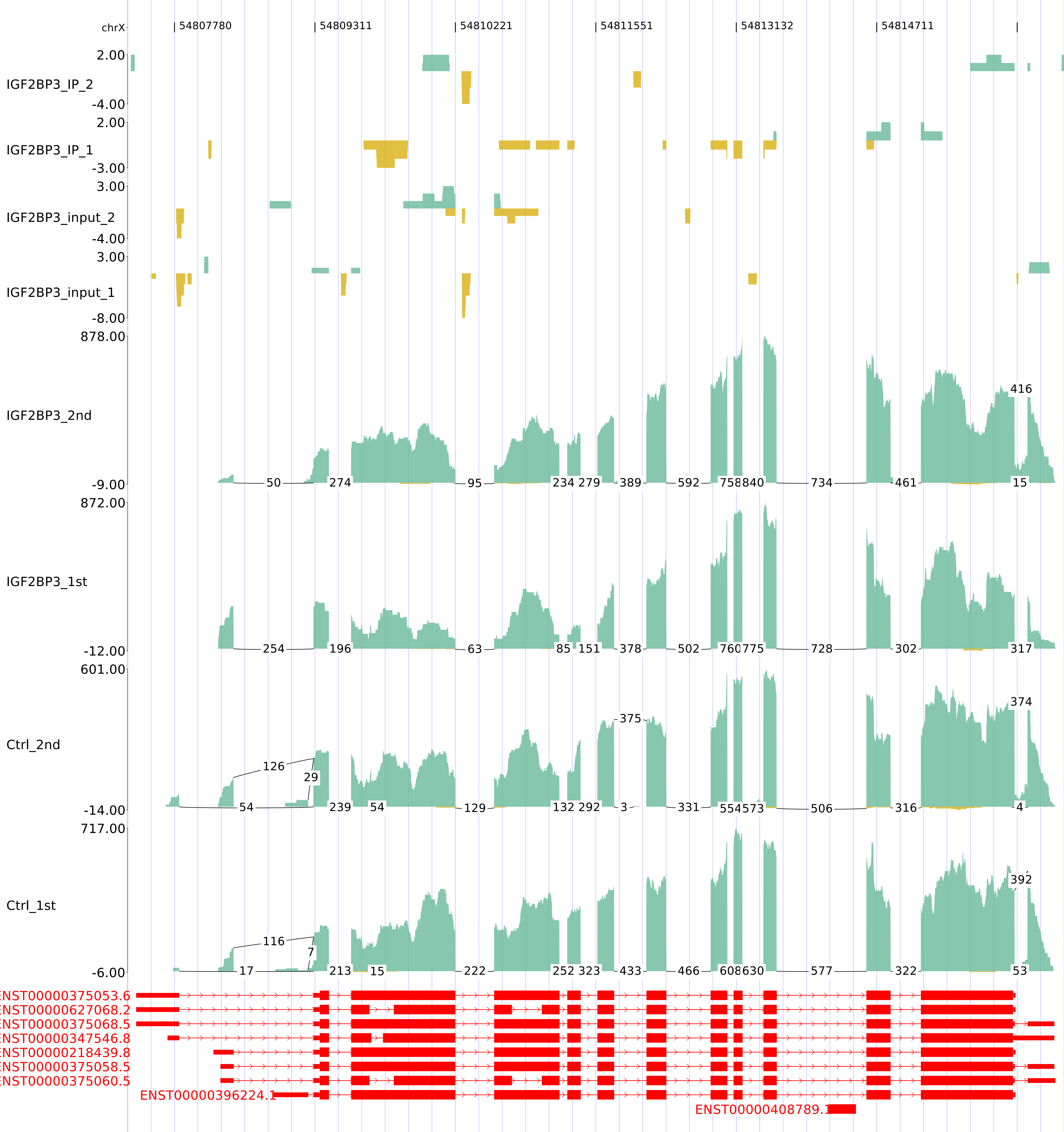

Supplement: Supplementary file 9 [file Data_Sheet_3.zip › ENSG00000102316.16_MAGED2.pdf]

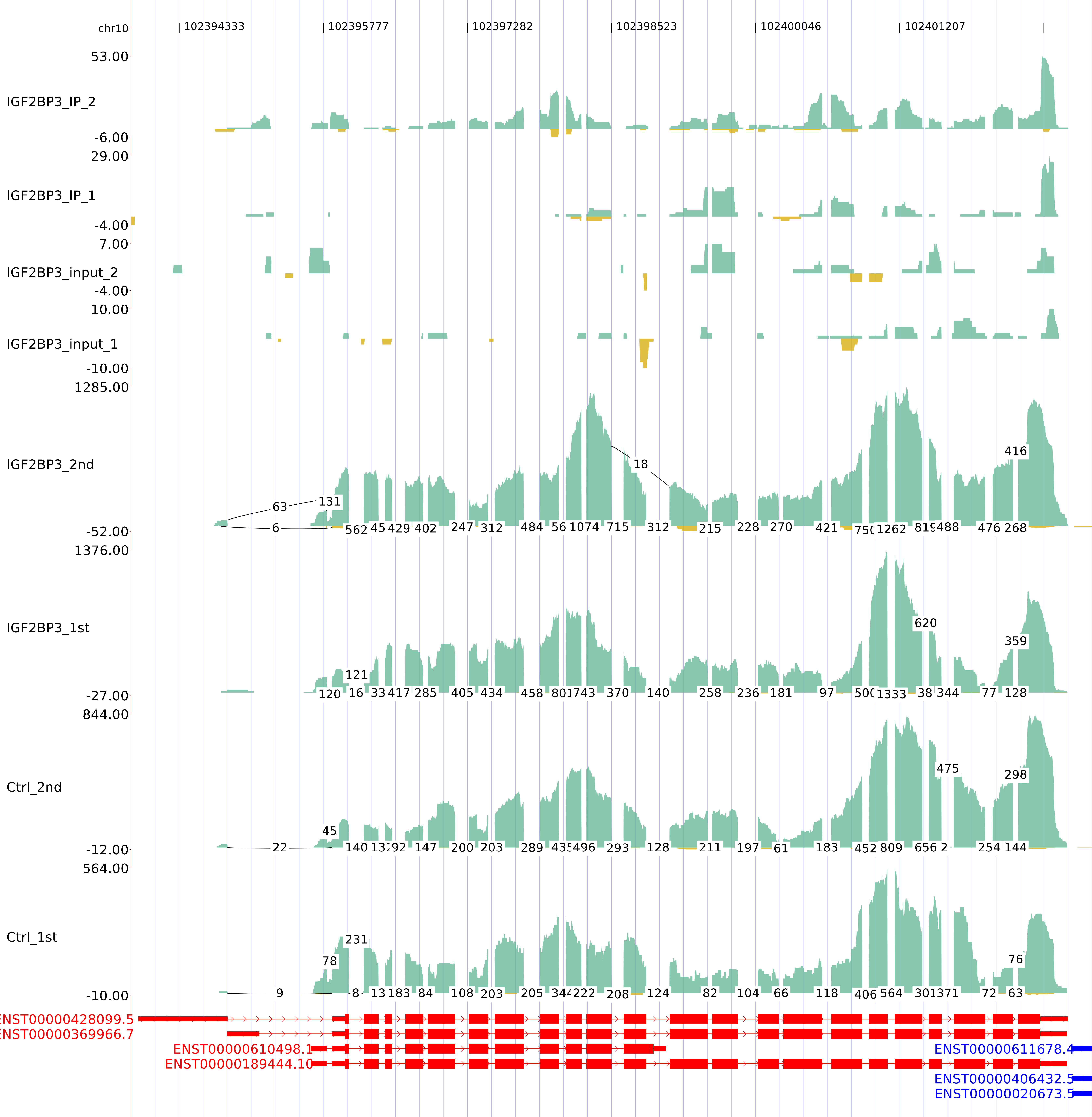

Supplement: Supplementary file 9 [file Data_Sheet_3.zip › ENSG00000077150.17_NFKB2.pdf]

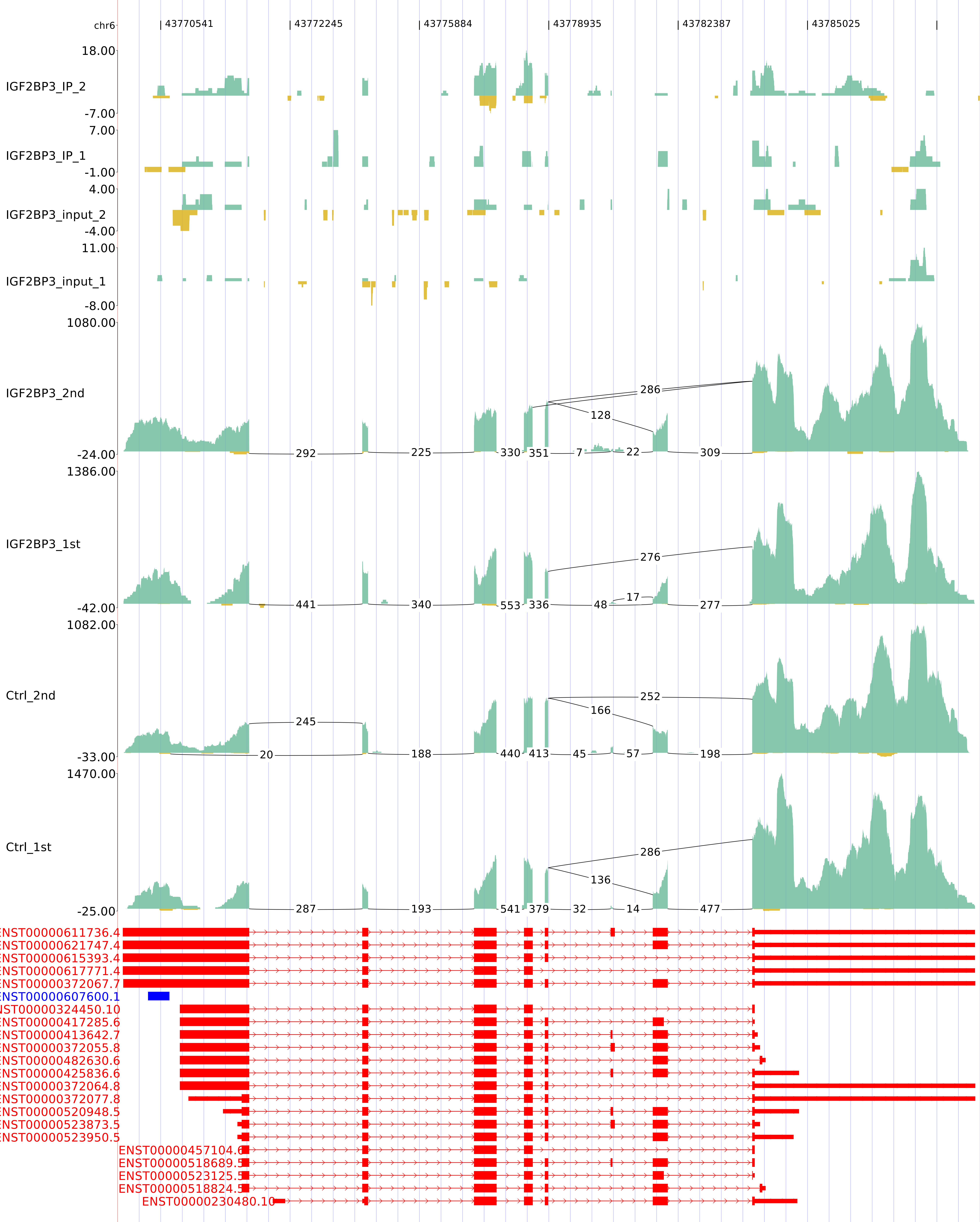

Supplement: Supplementary file 9 [file Data_Sheet_3.zip › ENSG00000112715.20_VEGFA.pdf]

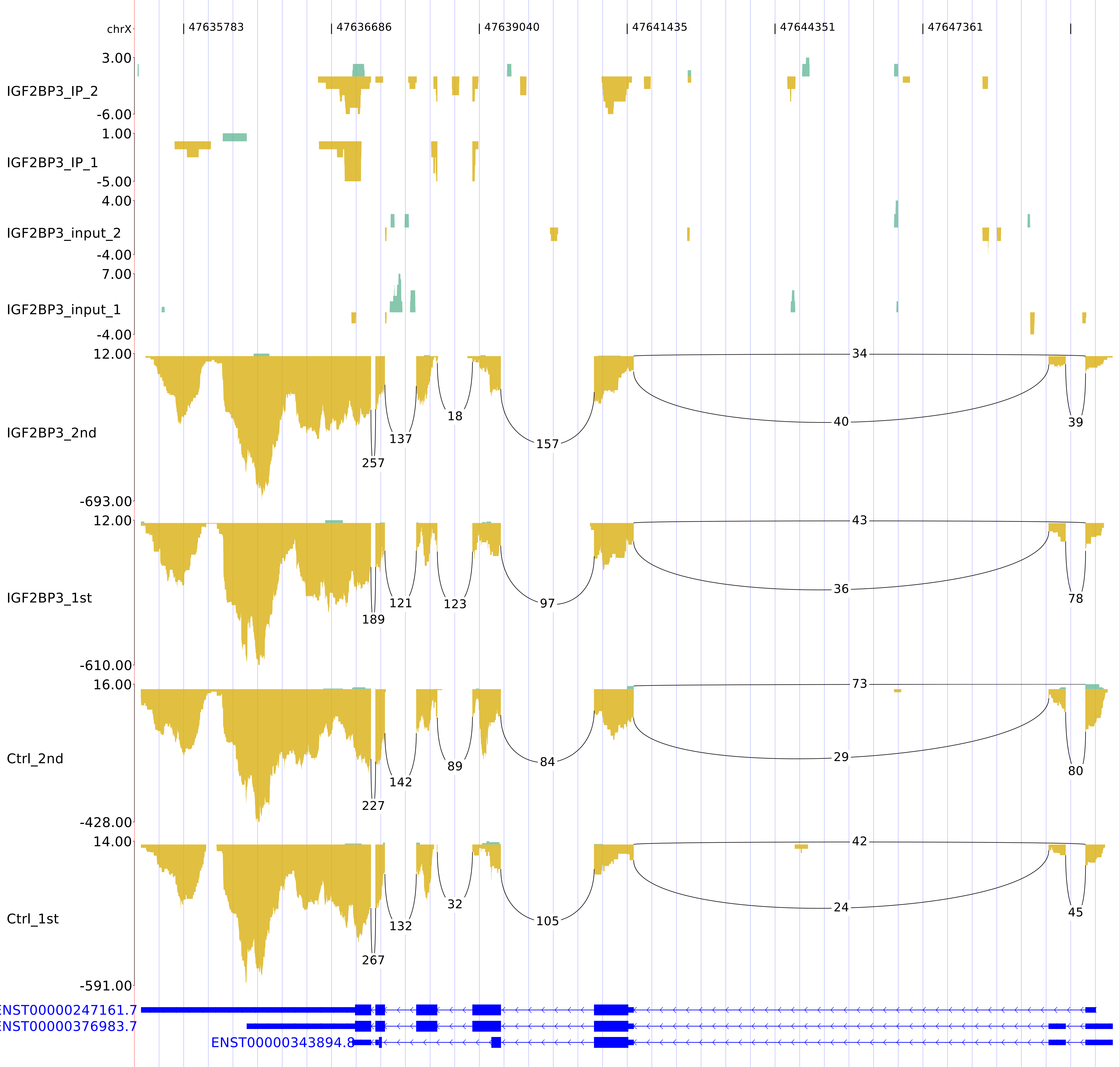

Supplement: Supplementary file 9 [file Data_Sheet_3.zip › ENSG00000126767.17_ELK1.pdf]

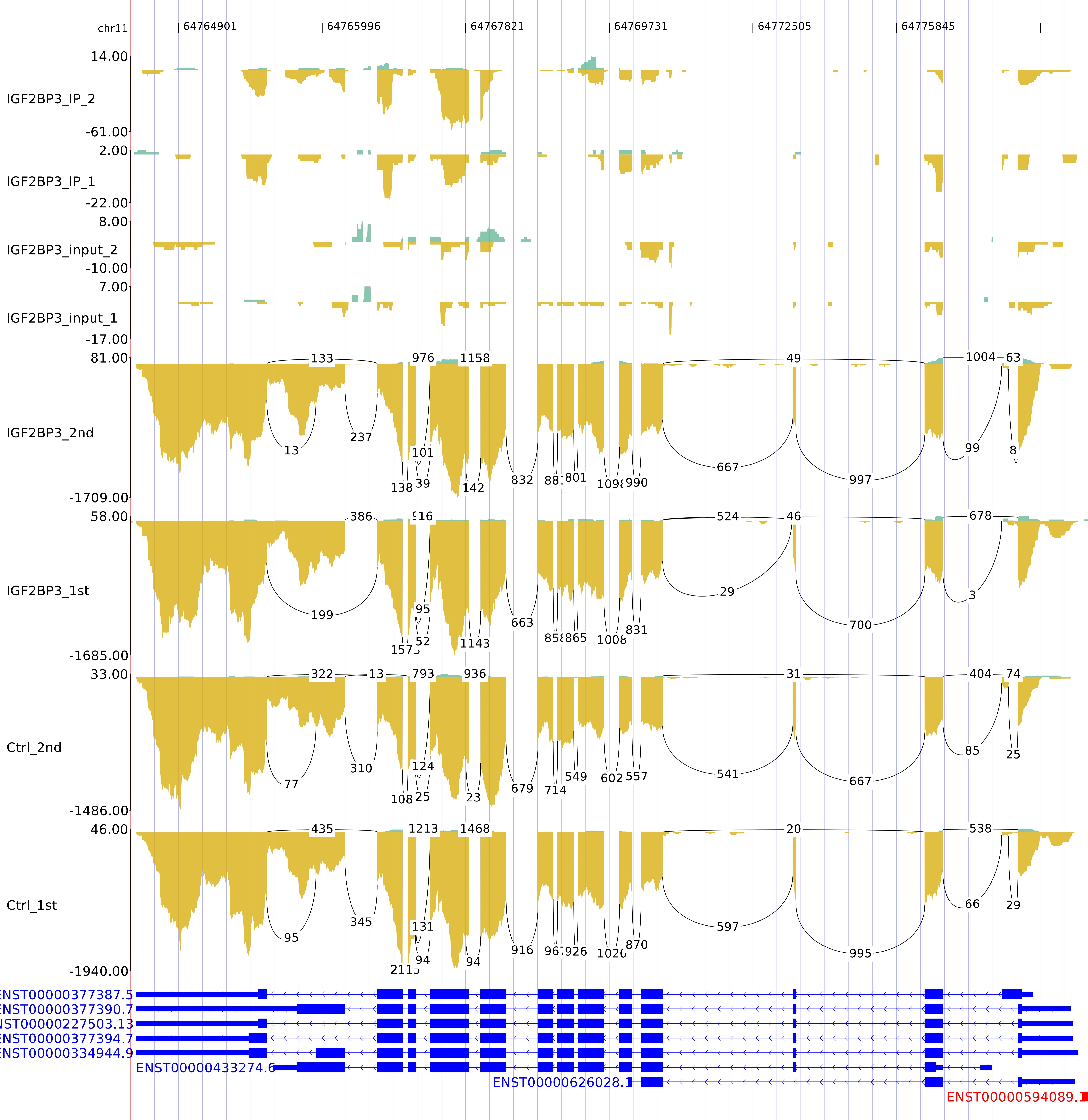

Supplement: Supplementary file 9 [file Data_Sheet_3.zip › ENSG00000168066.20_SF1.pdf]

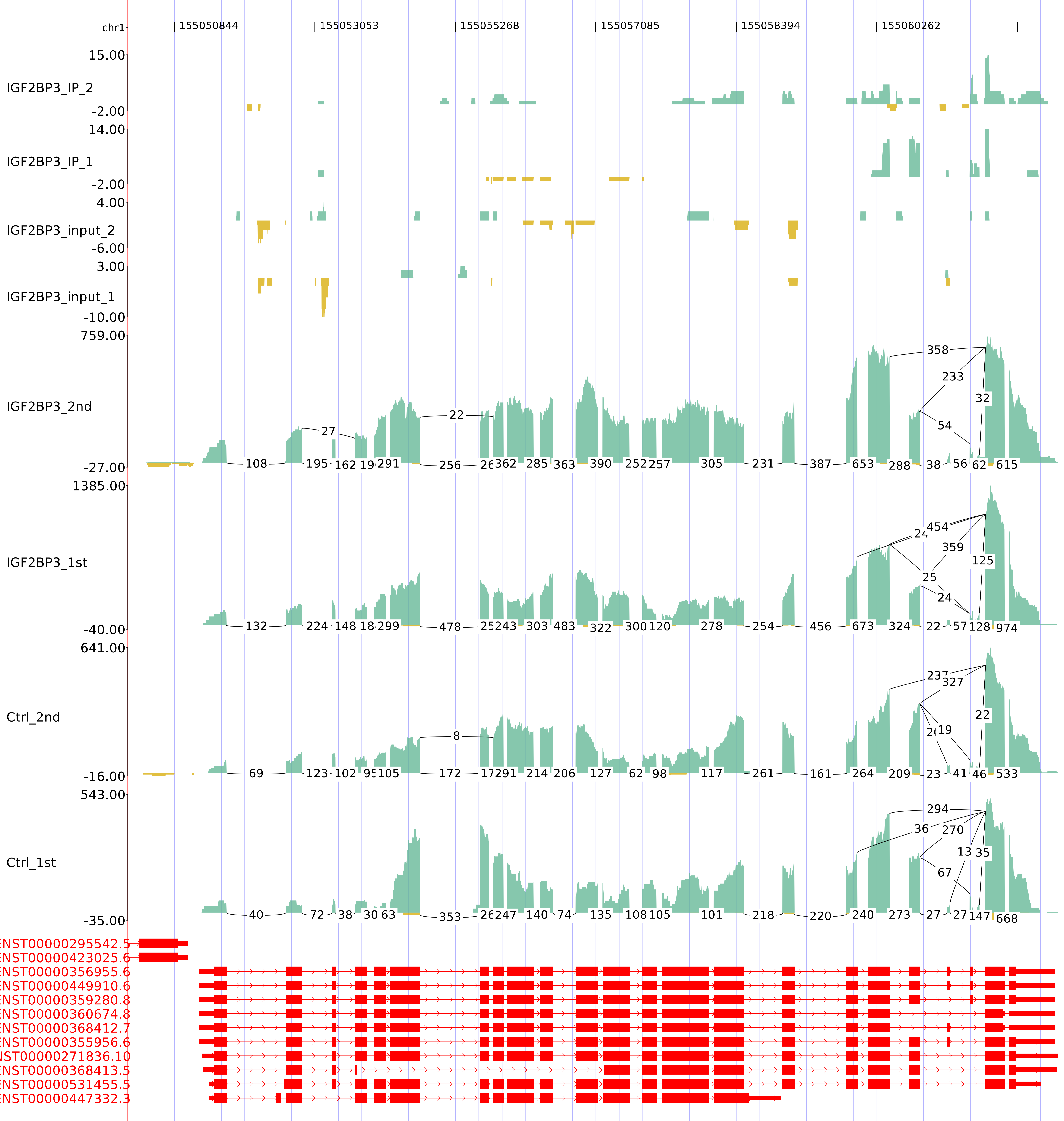

Supplement: Supplementary file 9 [file Data_Sheet_3.zip › ENSG00000143537.13_ADAM15.pdf]

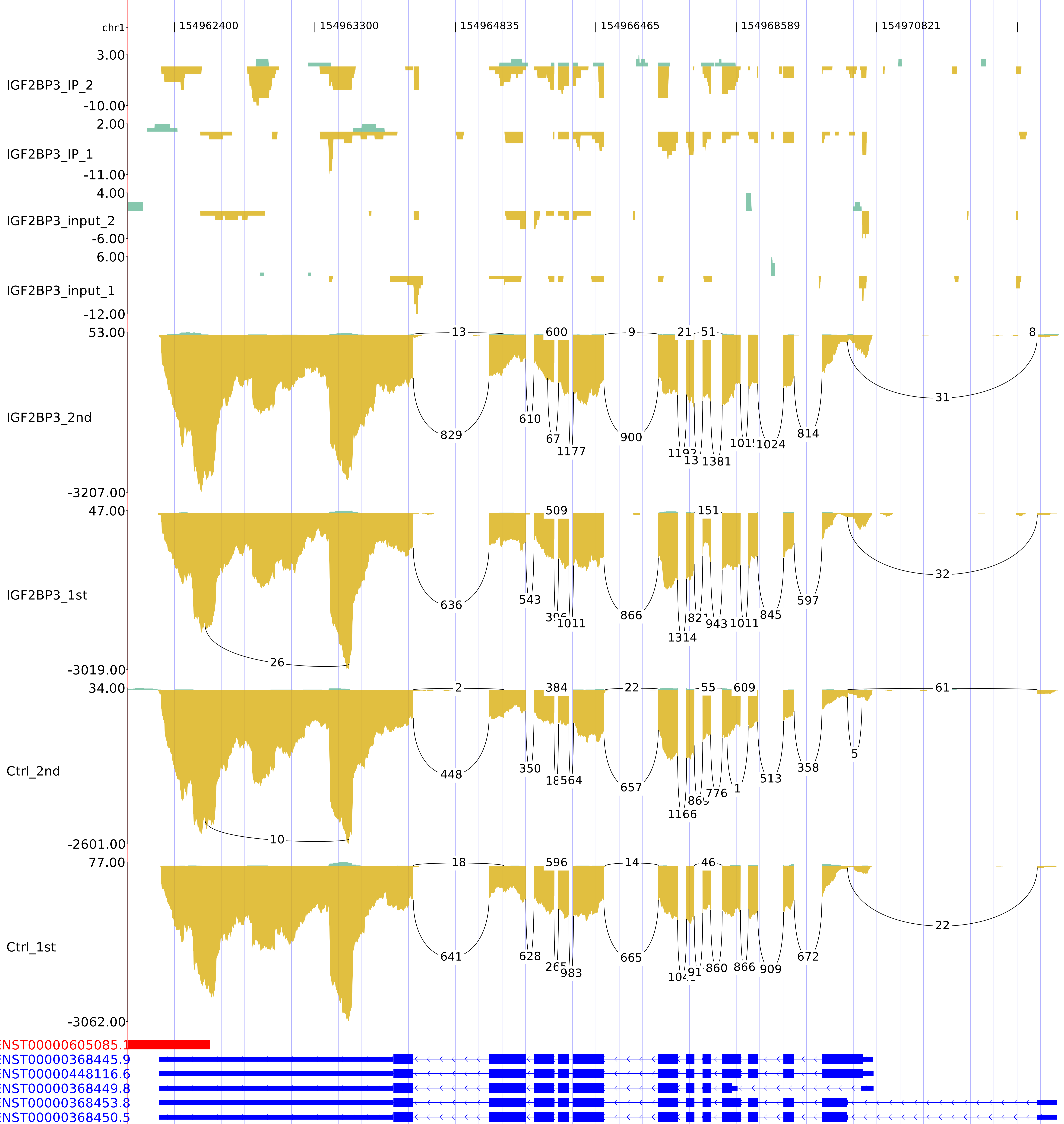

Supplement: Supplementary file 9 [file Data_Sheet_3.zip › ENSG00000160691.18_SHC1.pdf]

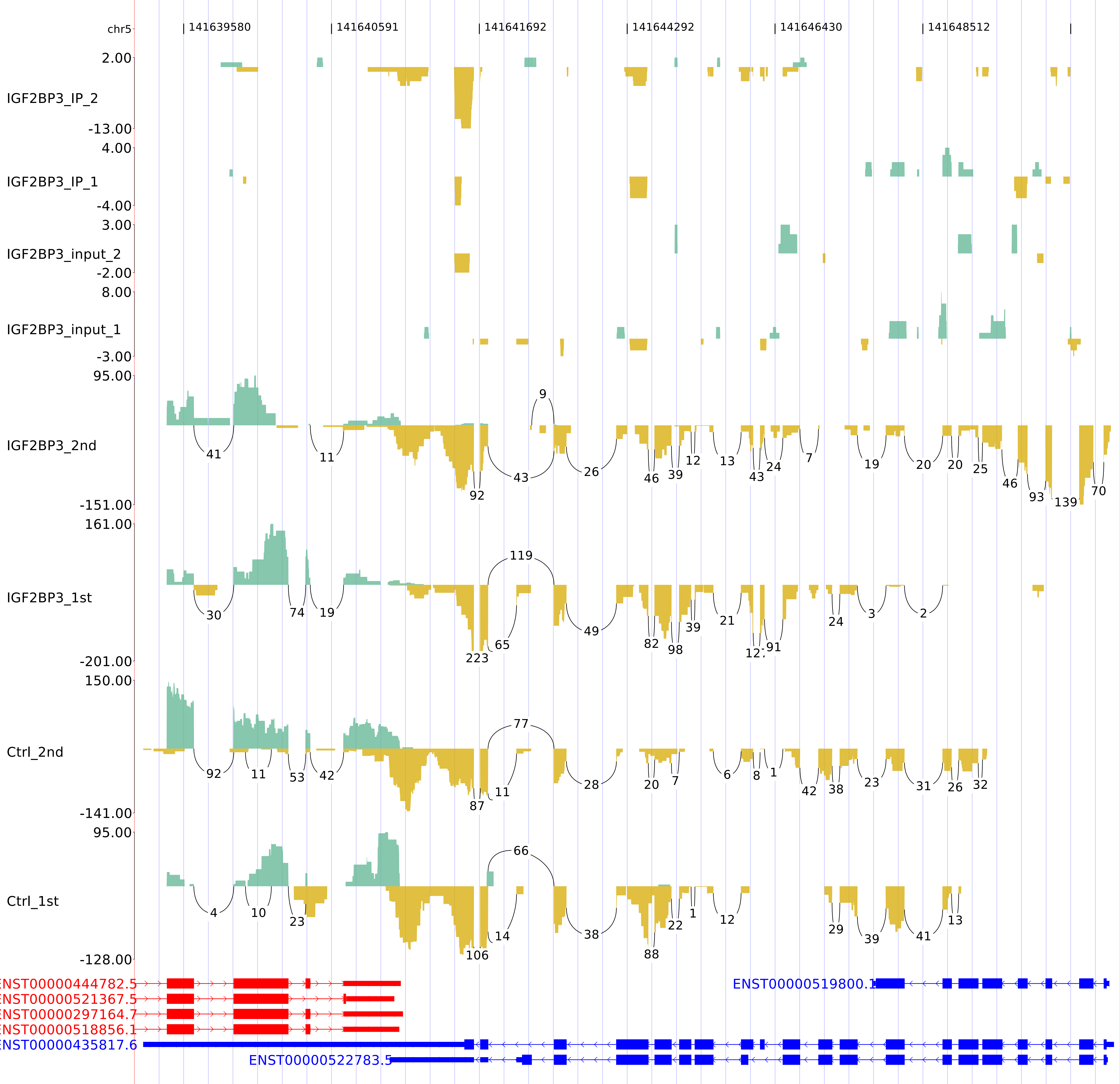

Supplement: Supplementary file 9 [file Data_Sheet_3.zip › ENSG00000197948.10_FCHSD1.pdf]

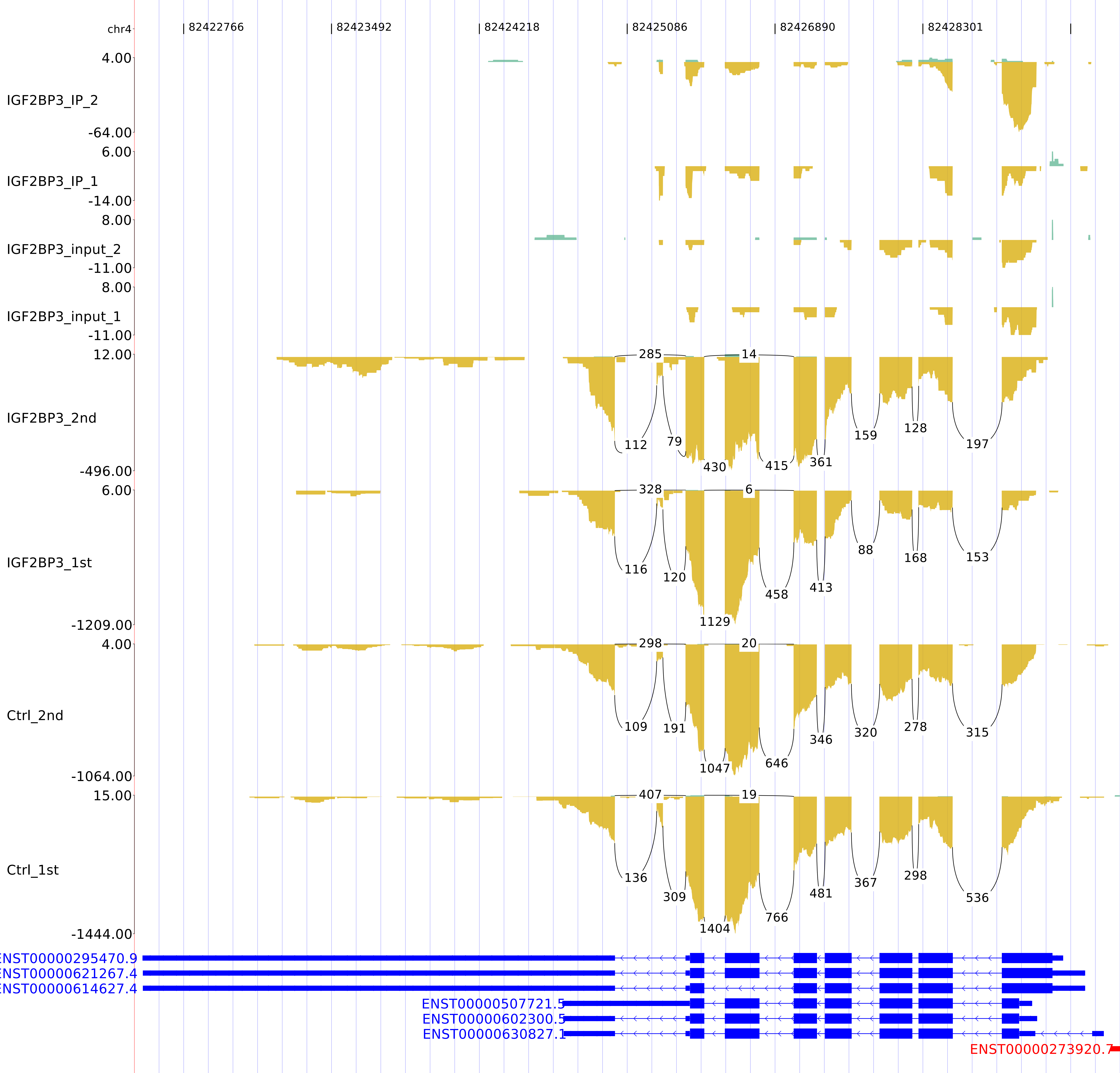

Supplement: Supplementary file 9 [file Data_Sheet_3.zip › ENSG00000152795.17_HNRNPDL.pdf]

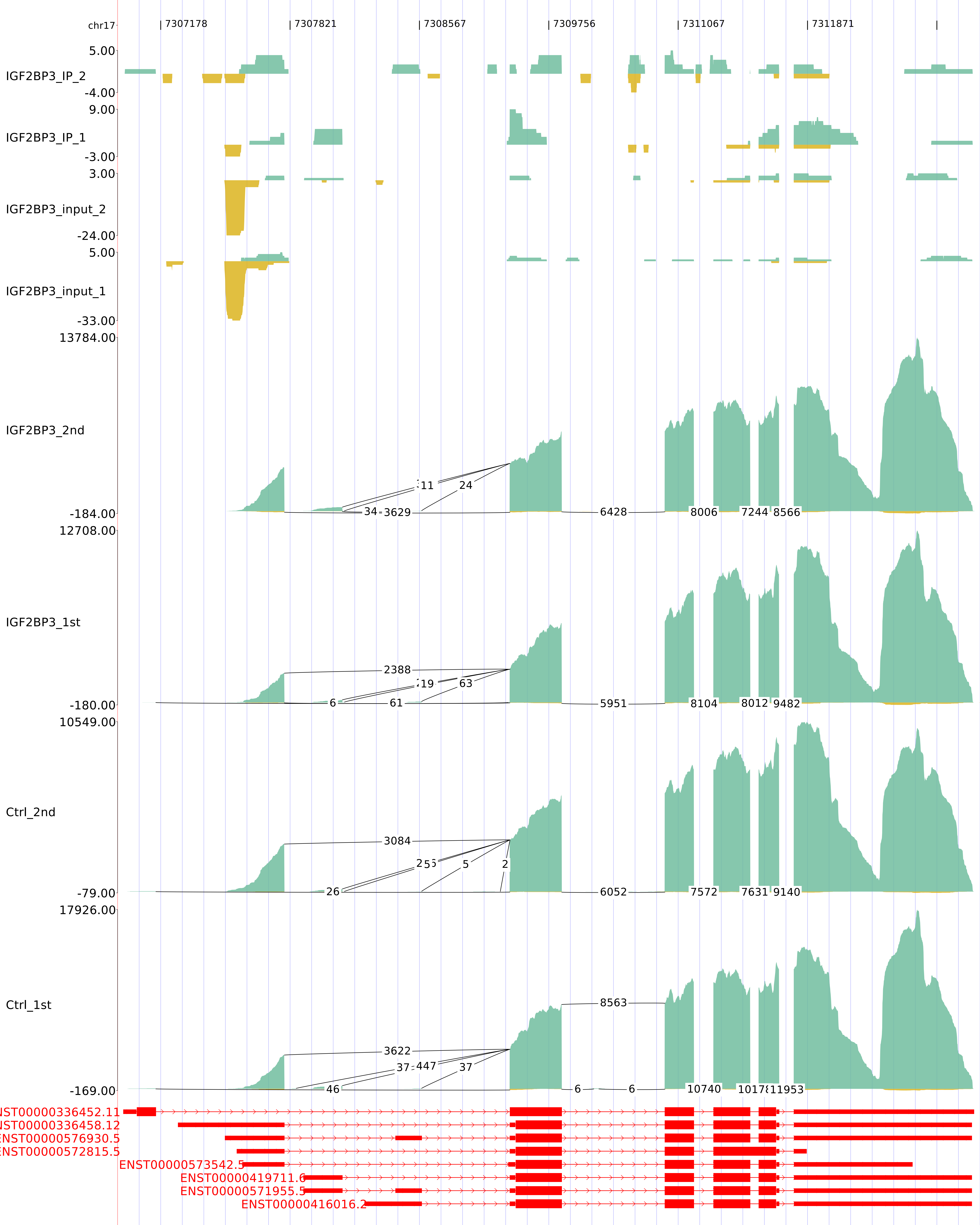

Supplement: Supplementary file 9 [file Data_Sheet_3.zip › ENSG00000132507.17_EIF5A.pdf]

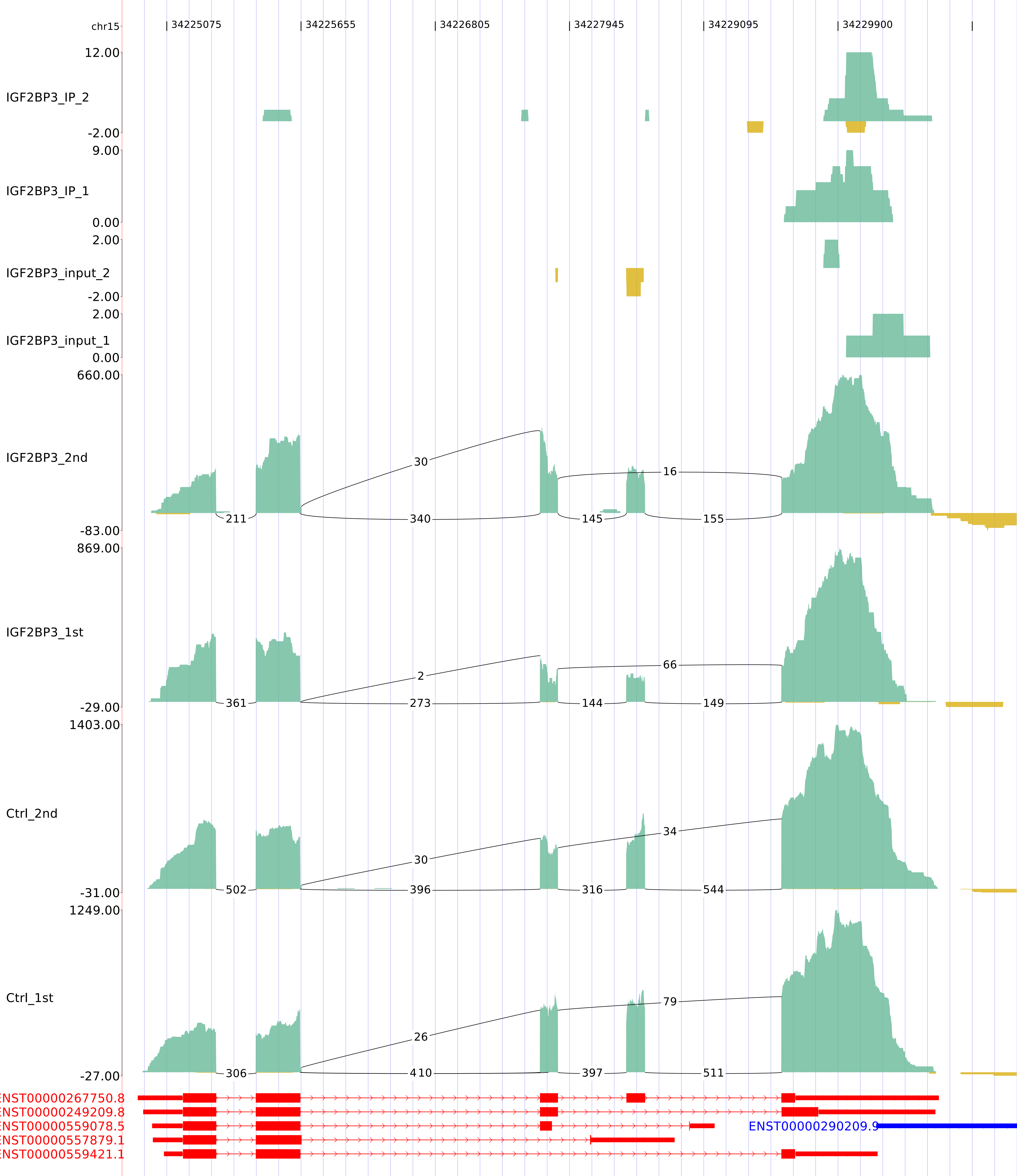

Supplement: Supplementary file 9 [file Data_Sheet_3.zip › ENSG00000128463.12_EMC4.pdf]

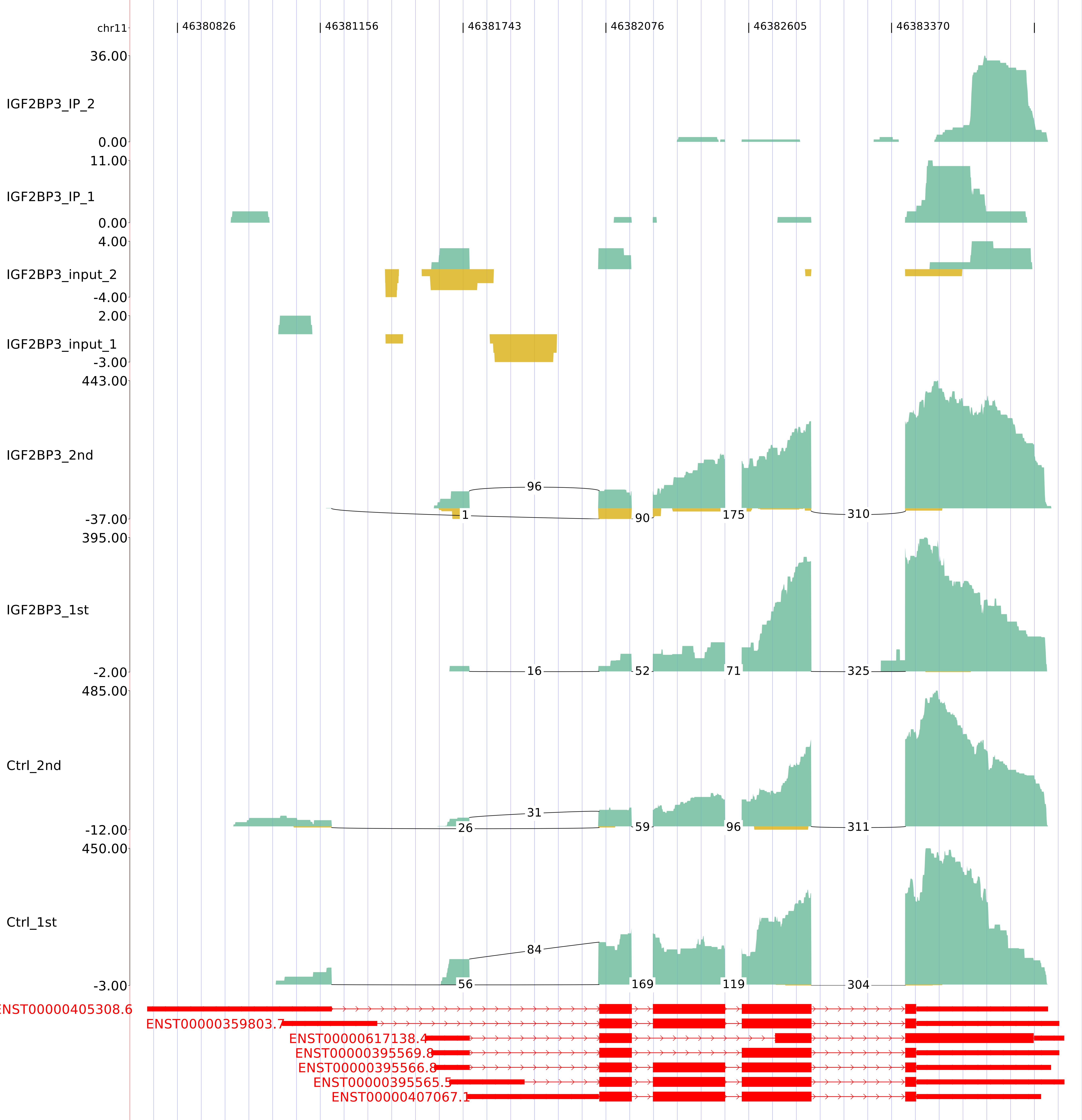

Supplement: Supplementary file 9 [file Data_Sheet_3.zip › ENSG00000110492.15_MDK.pdf]
